# Pulmonary Tuberculosis in the 19th Century: A Historical Case Study of Dr. Șerban Eminovici, Romanian Physician and Brother of Poet Mihai Eminescu

**DOI:** 10.3390/pathogens14101067

**Published:** 2025-10-21

**Authors:** Andrei Ionut Cucu, Catalin M. Buzduga, Navena Widulin, Alexandru Nemtoi, Amelian Madalin Bobu, Claudia Florida Costea, Roxana Filip, Vlad Porumb, Anca Petruta Morosan, Alexandru Carauleanu, Anca Sava, Elena Porumb-Andrese, Emilia Patrascanu, Camelia Tamas, Andreas G. Nerlich

**Affiliations:** 1 Faculty of Medicine and Biological Sciences, Stefan cel Mare University of Suceava, 720229 Suceava, Romania; andrei.cucu@usm.ro (A.I.C.); alexandru.nemtoi@usm.ro (A.N.); roxana.filip@usm.ro (R.F.); 2 Faculty of Medicine, Grigore T. Popa University of Medicine and Pharmacy Iasi, 700115 Iasi, Romania; claudia.costea@umfiasi.ro (C.F.C.); vlad.porumb@umfiasi.ro (V.P.); ale.carauleanu@umfiasi.ro (A.C.); sava.anca@umfiasi.ro (A.S.); elena.andrese1@umfiasi.ro (E.P.-A.); patrascanu.emilia@umfiasi.ro (E.P.); camelia.tamas@umfiasi.ro (C.T.); 3 Berlin Museum of Medical History, Charité—Universitätsmedizin Berlin, Charitéplatz 1, 10117 Berlin, Germany; navena.widulin@charite.de; 4 Clinical Emergency Hospital St. Spiridon, 700111 Iasi, Romania; amelian.bobu@gmail.com; 5 Institute of Legal Medicine, Department of Forensic Histology, Paleopathology and Mummy Research, Ludwig-Maximilians-University, 80336 Munich, Germany; andreas.nerlich@extern.lrz-muenchen.de

**Keywords:** pulmonary tuberculosis, 19th century medicine, Șerban Eminovici, Charité Hospital Berlin

## Abstract

**Background:** In the 19th century, pulmonary tuberculosis was the leading cause of death in Europe, responsible for up to one-quarter of all mortality. Before Robert Koch’s discovery of the tubercle bacillus in 1882 and the advent of effective therapies, treatment relied on rest, high-caloric diets, and sanatoria. **Objectives:** This study aims to reconstruct the medical biography of Dr. Șerban Eminovici (1841–1874), Romanian physician and elder brother of poet Mihai Eminescu, and to contextualize his life and death within the broader history of tuberculosis and pre-antibiotic medical practice. **Methods:** We conducted a historical case study using archival sources, including university registers from Erlangen, Munich, and Vienna, hospital admission records from the Charité Hospital in Berlin, and contemporaneous correspondence. Secondary literature on the history of tuberculosis and the Eminovici family was also reviewed. **Results**: Eminovici pursued medical studies across Central Europe, obtaining his doctorate in Vienna and later practicing medicine in Berlin, where he was a member of the Berliner Medizinische Gesellschaft. Despite early signs of respiratory illness, treated at spa resorts such as Gleichenberg, his condition progressed to advanced pulmonary tuberculosis with neuropsychiatric complications. Hospital records confirm his admission to the Charité on 10 October 1874, and his death from “Lungenschwindsucht” (pulmonary tuberculosis) on 29 November 1874, at age 33. His trajectory illustrates both the transnational mobility of Romanian intellectual elites and the therapeutic limitations of pre-antibiotic medicine. **Conclusions:** The case of Dr. Șerban Eminovici highlights the devastating impact of tuberculosis on 19th-century intellectuals, the reliance on lifestyle-based therapies before the discovery of the tubercle bacillus, and the importance of Central European medical networks in shaping Romanian professional identities. Beyond its biographical significance, this case underscores the persistent social and cultural burden of tuberculosis in Eastern Europe.

## 1. Introduction

In the 19th century, pulmonary tuberculosis was the leading cause of mortality in Europe, being responsible for up to one-quarter of all deaths. The disease, often referred to as the “robber of youth,” disproportionately affected young people and lower socio-economic classes, accounting for one in four deaths in both Europe and the United States during that century [1,2]. Long before Robert Koch’s discovery of the tubercle bacillus in 1882 and the advent of antibiotics, pulmonary tuberculosis was treated through rest and balanced diets, as well as stays in sanatoria, which were established across Europe following the German model introduced by the physician Hermann Brehmer in 1854 [3], or through isolation of contagious patients. Essentially, pre-antibiotic treatment approaches to tuberculosis were focused on lifestyle modifications, supportive care, and nursing.

**Figure 1:**
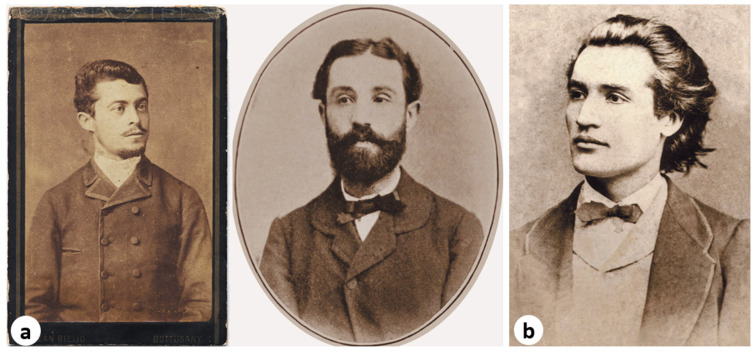
(**a**) Dr. Șerban Eminovici (1841–1874), (**b**) his brother, the national poet of the Romanians, Mihai Eminescu (1850–1889) *(reproduced with the permission of the Memorial Ipotești—National Centre for Mihai Eminescu Studies)*.

The present study draws attention to such a case, that of Dr. Șerban Eminovici (1841–1874), a Romanian physician trained in Central Europe, whose professional trajectory ended with his death from pulmonary tuberculosis at the Charité Hospital in Berlin. Șerban Eminovici (Figure 1a) was the elder brother of Mihai Eminescu (1850–1889) (Figure 1b), considered the greatest Romanian poet of all time.

## 2. Archival Sources and Methodology

Archival sources were identified through systematic searches conducted in institutional collections and digital repositories in Romania, Austria, and Germany. The primary corpus included:

(1) University registers and matriculation books from the Universities of Erlangen, Munich, Vienna, and Berlin;
(2) Hospital admission and death records from the Charité Hospital in Berlin *(courtesy of Navena Widulin from the Berlin Museum of Medical History at the Charité)*
(3) Personal and diplomatic correspondence preserved at the Romanian National Archives and the Memorial Ipotești—National Centre for Mihai Eminescu Studies.

Each document was authenticated by cross-referencing institutional catalog numbers, seals, signatures, and dates with the official archival inventories.

Inclusion criteria required: (1) clear provenance from an institutional archive or published primary collection, (2) direct biographical relevance to Șerban Eminovici or his immediate family and (3) verifiable authenticity (archival marks, registry codes, or publication reference). Exclusion criteria included: (1) documents of uncertain origin or incomplete metadata, (2) later secondary reproductions without verifiable primary reference, and (3) anecdotal or non-sourced historical accounts.

Secondary literature [4,5,6,7,8,9,10,11] was used only to contextualize verified primary data and was critically compared with archival findings.

## 3. Background, Mortality Patterns, and Health Context in the Eminovici Family

**Figure 2:**
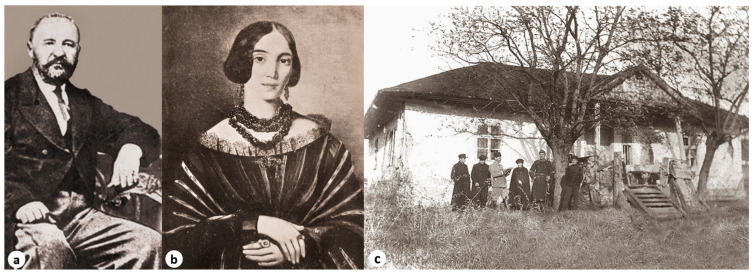
(**a**) Gheorghe Eminovici (1812–1884), father of Șerban Eminovici, (**b**) Raluca Eminovici (1816–1876), mother of Șerban Eminovici, (**c**) the family house in Ipotești, where Șerban Eminovici was raised and spent his childhood *(reproduced with the permission of the Memorial Ipotești—National Centre for Mihai Eminescu Studies)*.

Șerban Eminovici was the first child of Gheorghe and Raluca Eminovici [12] (Figure 2a,b), and although he was born in Dumbrăveni (Romania) in 1841, he spent most of his childhood in Ipotești, where his father, Gheorghe Eminovici, had purchased an estate and a boyar’s house (Figure 2c). Serban was born to a family affected by tuberculosis and predisposed to mental disorders [13].

**Figure 3:**
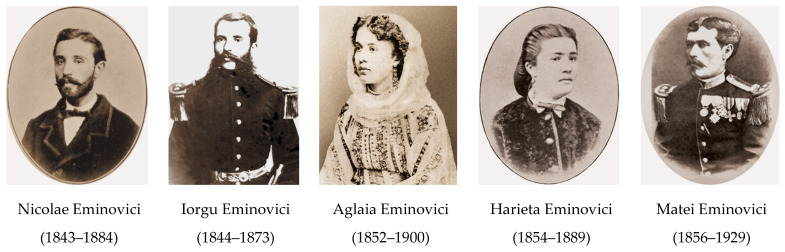
Some of Șerban Eminovici’s siblings who survived into adulthood *(reproduced with the permission of the Memorial Ipotești—National Centre for Mihai Eminescu Studies)*.

At birth, Șerban Eminovici was described as “dark-skinned, slender, with narrow shoulders” [5], “quiet, morose, yet with a handsome appearance, resembling his mother’s side” [4]. Șerban Eminovici was the first child of the Eminovici family and was followed at short intervals by his siblings, some of whom died prematurely: Nicolae (1843–1884), Iorgu (1844–1873), Ilie (1846–1867), Maria (1848–1856), Mihai (1850–1889), Aglaia (1852–1900), Ruxandra (1854–1854), Harieta (1854–1889), Matei (1856–1929), and Vasile (1856–1860) (Figure 3).

A defining characteristic of nearly all the children of Gheorghe and Raluca Eminovici was their remarkable intelligence, many of them pursuing careers in the military, medicine, as well as in the arts and culture [12].

Of Șerban Eminovici’s ten siblings, three died at very young ages: Ruxandra Eminovici (b. 1854–d. 1854) passed away before the age of one; Maria Eminovici (1848–1856) died at the age of eight from an unknown childhood illness; and Vasile Eminovici (1856–1860) also died at the age of three. Several of his other siblings died young, among them two brothers (Nicolae and Iorgu) who both committed suicide by gunshot [8,14]. His sister Aglaia died of tuberculosis, and another brother, Matei, likely suffered from a chronic pulmonary disease, although the exact diagnosis remains uncertain [4,5,6,7,8,13,14] (Table 1).

Their mother, Raluca Eminovici, died at the age of 60 in 1876 from cancer [8], while their father, Gheorghe Eminovici, passed away in 1884 at the age of 72 due to a prostatic disorder [8]. The average age at death among the Eminovici children was 30.1 years, with Matei being the most long-lived, dying at the age of 73 (Table 1).

In Western Europe, and consequently in Romania, during the period 1880–1900, the average life expectancy at birth was approximately 35 years [15]. In the mid-nineteenth century, within the Austrian Empire, the region where Șerban Eminovici pursued his studies, life expectancy was similarly low, with estimates ranging between 30 and 36 years for men [16]. During that period, the main causes of mortality in Europe were tuberculosis and other infectious diseases such as measles, typhus, and cholera. Tuberculosis accounted for a significant share of mortality across the continent [17], with estimates suggesting that it was responsible for one in every four deaths in the nineteenth century [18].

## 4. Educational Trajectory and Medical Training of Dr. Șerban Eminovici

In 1852, the young Șerban was already enrolled in the third grade at the Ladislav Ferderber Boarding School in Botoșani, where he studied alongside his brothers, and later continued at the German Lyceum in Cernăuți (Ober Lyceum) [9]. On April 2, 1852, Șerban Eminovici was listed in the March catalog of the Ladislav Ferderber Boarding School in Botoșani, together with his brother Nicolae [19]. In the same year, on August 23, Șerban’s father, Gheorghe Eminovici, requested from the State Secretariat the issue of a passport for his sons, Șerban and Nicolae, who were to pursue their studies together in Cernăuți, the age difference between them being two years [19]. His academic record at the German Lyceum in Cernăuți was modest, with low general grades and repeated classes [20].

**Figure 4:**
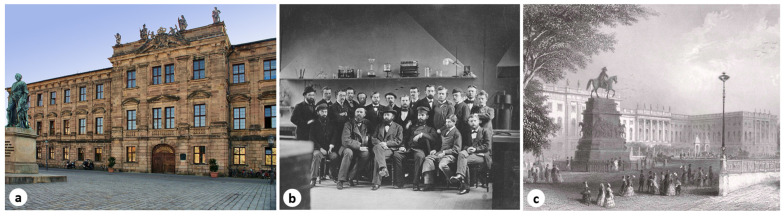
(**a**) Königlich Bayerische Friedrich-Alexander Universität *[Schloss Erlangen (Selby, CC BY-SA 3.0*, via *Wikimedia Commons)]*, (**b**) photograph taken in 1877 depicting chemistry professors and doctoral students during the winter semester 1877/1878, including Adolf von Baeyer, Emil Fischer, and Jacob Volhard, at the Ludwig Maximilian University of Munich *(public domain)*, (**c**) the University of Berlin during Șerban Eminovici’s student years *(public domain)*.

A few years later, in 1864, Șerban Eminovici was among the students of the National School of Agriculture in Pantelimon, the precursor of the future Institute of Agronomy in Bucharest (Romania) [9]. Uninterested in a career in agronomy, Șerban turned to his earlier vocation, that of becoming a physician. Thus, on November 30, 1865, he enrolled at the Faculty of Medicine in Erlangen (Figure 4a), where he attended only four semesters until December 2, 1867, after which he continued with one more semester at the University of Munich (Figure 4b), and then at the University of Berlin (Figure 4c), where he practiced with distinction as a member of the “Scientific-Medical Society” [10].

The Royal Bavarian Friedrich-Alexander University of Erlangen (Figure 4a) had, in the academic year 1867–1868, nine faculties with multiple specializations. Within the Faculty of Medicine, there were three specializations: General Medicine, Surgery, and Pharmacy, with a total of 225 students, 35 of whom were foreigners. Among the Romanian students were Herrmann Johan Carl from Turnu Măgurele, enrolled in the Faculty of Chemistry, and Engel Johan from Iași, enrolled in the Faculty of Pharmacy. According to the research of Dulciuc, Serban Eminovici was listed in the Yearbook of the Royal Bavarian University of Erlangen for the winter semester 1865–1866. He had also been officially enrolled on 30 November 1865, under matriculation number 76. At that time, Șerban Eminovici (Eminovicz, Scherban) resided at Karlstrasse no. 376, lodging with a shoemaker named Hartmann [9].

**Figure 5:**
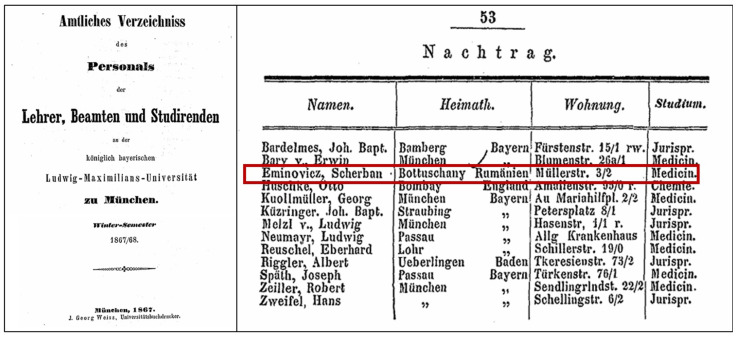
Șerban Eminovici (Scherban Eminovicz) listed as a medical student in the supplement (Nachtrag) to the professors’ and students’ catalog of the Ludwig Maximilian University of Munich, Winter Semester 1867/1868 *(image courtesy of Prof. Dr. med. Dr. rer. hum. biol. Andreas G. Nerlich)*.

In a supplement (Nachtrag) to the professors’ and students’ catalog, Șerban Eminovici is recorded as a student in the winter semester of 1867/1868 at the Faculty of Medicine of the Ludwig Maximilian University of Munich (Figure 5), with his residence listed at Müllerstrasse 3/2 [9].

**Figure 6:**
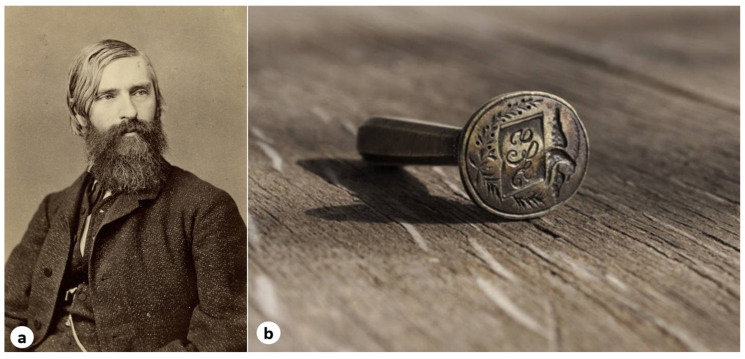
(**a**) Portrait of Albrecht von Graefe (1828–1870), the first president of the Berlin Medical Society, during the period 1860–1870, when Dr. Șerban Eminovici was a member of this society (Photograph by H. Joop, Berlin, 1870) *(public domain)*; (**b**) seal of Dr. Serban Eminovici with his initials “S” and “E.” *(reproduced with the permission of the Memorial Ipotești—National Centre for Mihai Eminescu Studies)*.

Consequently, Șerban appears in the Yearbook of the Faculty of Medicine in Erlangen for the academic years 1865–1867, while in the Yearbook of the Ludwig Maximilian University of Munich he is listed as enrolled for the academic year 1867–1868. During 1867–1868, Serban was also registered as a doctoral candidate in medicine at the University of Munich. In this period, his brother Mihai Eminescu wrote to their father in Romania, stating “that in the capital of Germany, his brother was already appreciated as a good physician, and that Șerban Eminovici was recognized for his professional competence and academic title” [11]. Ultimately, Serban became a physician and was respected by those around him, becoming a member of a scientific-medical society in Berlin (Berliner Medizinische Gesellschaft), which reflected his recognition within the medical community of the time (Figure 6).

In the autumn of 1869, the young Șerban Eminovici was in Prague, where he lived together with his brother, the poet Mihai Eminescu, in an apartment on Lipova Street no. 471/6, near the Faculty of Medicine [10]. Around the same years, he also resided in Vienna, where he lived close to the old Allgemeiens Krankenhaus (AKH Hospital), on Alserstrasse no. 25 [10]. The location of these residences reflects Șerban Eminovici’s preference for choosing lodgings in the vicinity of medical clinics. Even in Berlin, he selected accommodations near the famous Charité Hospital [10].

**Figure 7:**
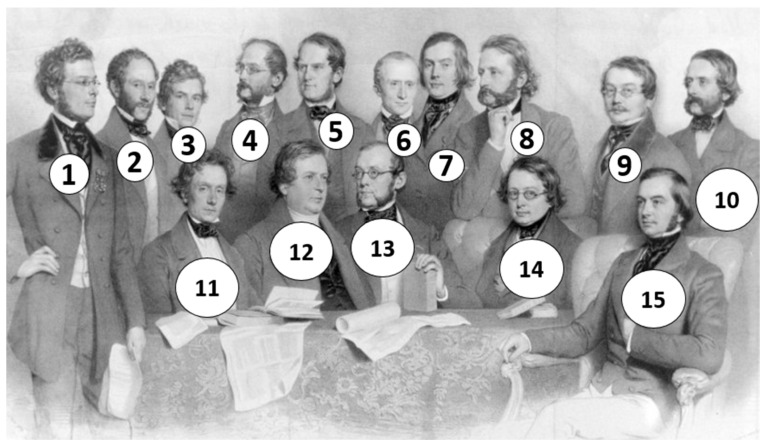
The Faculty of Medicine Professors’ Council, University of Vienna, during the student years of Serban Eminovici (1853). Standing, from left to right: ①—Josef Hyrtl, ②—Karl Ludwig Sigmund von Ilanor, ③—Joseph Redtenbacher, ④—Franz Unger, ⑤—Carl Haller, ⑥—Ernst Wilhelm von Brücke, ⑦—Johann Ritter von Oppolzer, ⑧—Theodor Helm, ⑨—Ferdinand von Hebra, ⑩—Johann Nepomuk H. Dlauhy. Seated, from left to right: ⑪—Franz Schuh, ⑫—Anton von Rosas, ⑬—Carl Freiherr von Rokitansky, ⑭—Josef Skoda, ⑮—Johann Heinrich Dumreicher *(Archiv der Universität Wien, Bildarchiv, Signature: 106.I.2139, public domain)*.

During his Viennese period, he lived on Alserstrasse no. 25, where his mentor, the Austrian physician Johann Ritter von Oppolzer (1808–1871), also had his practice (Figure 7, no. 7) [9]. In Vienna, he attended all the regular courses taught by leading professors (Figure 7) and successfully passed his doctoral examinations, being described as a “brilliant student” [4]. Given Oppolzer’s specialty in electrotherapy, Eminovici’s interest in acquiring a faradization apparatus can be understood [21]. On July 28, 1871, he was awarded the title of “doctor” by the University of Vienna [12].

## 5. Early Signs of Illness and Sanatorium Treatments in the Habsburg Empire

**Figure 8:**
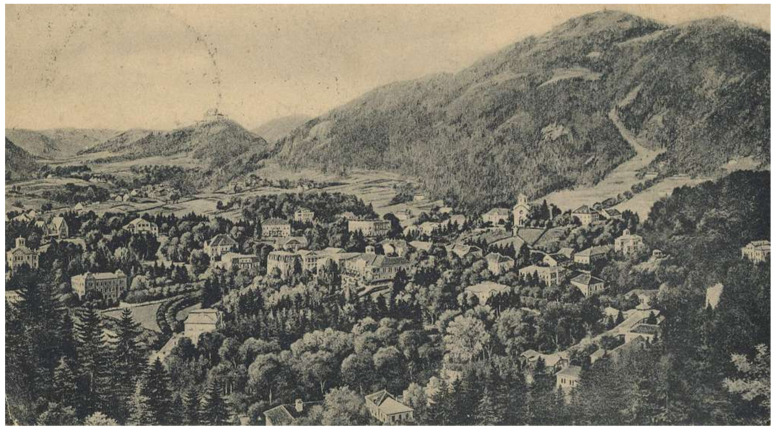
General view of the Gleichenberg balneary resort during the period when Serban Eminovici received treatment for his respiratory illness there *(public domain)*.

As an adolescent, Serban was of frail health; therefore, in 1865, while studying at the Faculty of Medicine in Erlangen, Serban Eminovici visited and underwent treatment at the Gleichenberg resort (Styria, Austria) (Figure 8), one of the most exclusive spa and health resorts of the Habsburg Empire at that time, most likely upon his physicians’ recommendation.

This resort was renowned for its sulfurous and ferruginous springs, known since the Middle Ages, as well as for its clean air. Balneary medicine was highly valued in the 19th century, and physicians often prescribed mineral water cures for digestive, rheumatic, and respiratory conditions [22,23]. At that time, the newspaper Grazer Abendpost regularly published lists of foreign visitors, and the Romanian Eminescu scholar Dulciu identified in the July 1, 1865 issue the name of Șerban Eminovici, a student from Botoșani, Moldova (“Scherban Eminowicz, Studierender, v. Bottuschan in der Moldau”) [9].

**Figure 9:**
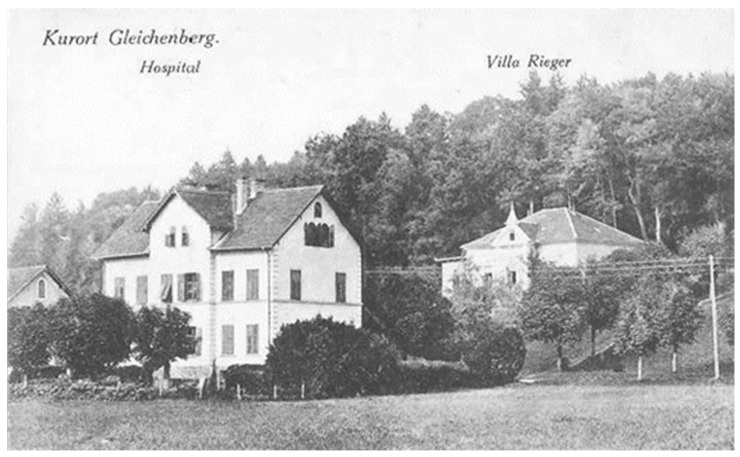
The sanatorium where Serban Eminovici stayed during his visits to the Gleichenberg balneary resort *(public domain)*.

Given his student status, Serban most likely opted for the cheapest accommodation, either lodging privately or staying at the sanatorium operated by the Gleichenberger Johannisbrunnen Actien-Verein, based on student medical insurance (Figure 9).

It was well known that student associations often covered part of the hospitalization expenses for young people who fell ill while studying abroad. In this context, it can be assumed that by the age of 24, Serban Eminovici was already suffering from a pulmonary disease serious enough to require repeated stays in various sanatoria across the Habsburg Empire.

At that time, tuberculosis posed a major public health threat in Europe. Knowledge about the etiology and treatment of the disease was extremely limited, and the German physician Hermann Brehmer, who in 1854 had established the first open-air sanatorium for tuberculosis in Germany, believed that the disease could be cured through a regimen of fresh air, physical exercise, and a nutritious diet in a forested environment [24].

In the autumn of 1872, Serban returned to his parents in Ipotești, his native village in Romania, to be treated by physicians in Botoșani, as he was gravely ill with pulmonary tuberculosis [10]. After unsuccessful treatments, he traveled back to Berlin to continue care for pulmonary tuberculosis at the renowned Charité Hospital, which was recognized for its advanced therapeutic approaches compared to most European hospitals [25]. Although it had originally been founded in 1710 to address public health crises such as plague, by the 19th century Charité had become a leading center for infection prevention as well as psychiatric treatment, shaping medical practice throughout Europe [26,27,28].

## 6. Final Stage of Illness and Hospitalization at the Charité, Berlin

**Figure 10:**
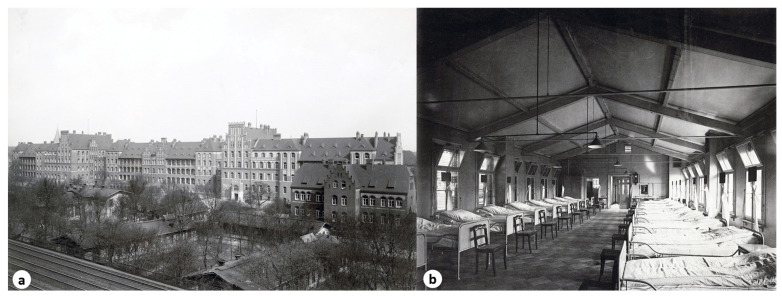
(**a**) Charité Medical Clinic I and II, Berlin *(Photo: Architectural Museum of the Technical University of Berlin, Inv. No. BZ-F 25.011)*, (**b**) Charité, hospital ward in the Institute for Infectious Diseases, around 1892 *(Photo: Hermann Rückwardt. Source: Architectural Museum of the Technical University of Berlin, public domain)*.

On October 10, 1874, Serban Eminovici was admitted to the Charité Hospital in Berlin with the support of the Romanian Agency in Berlin (Rumänischen Gesandtschaft zu Berlin) [10] (Figure 10).

The Charité Hospital in Berlin, founded in 1710, was one of the most prestigious medical institutions in Europe during the 19th century. Nevertheless, at that time, treatment for pulmonary tuberculosis was limited, preceding the antibiotic era, and relied on rest, adequate nutrition, and fresh air. Șerban Eminovici’s admission to the Charité reflects both the severity of his illness and the hope placed in the advanced medical care provided by this institution [1].

In the second half of the 19th century, the treatment of pulmonary tuberculosis at the Charité Hospital in Berlin reflected the prevailing pre-antibiotic medical paradigms. Patients, including Șerban Eminovici, brother of the poet Mihai Eminescu, admitted in October 1874, benefited from a regimen based on strict physical rest, high-protein diets, and constant exposure to fresh air, all considered essential for “strengthening” the body in its fight against the disease [1,28]. Efforts were also directed toward maintaining a calm and hygienic environment, within a medical framework emphasizing isolation, rigorous clinical observation, and the use of tonic remedies such as cod liver oil, opiates, or small doses of arsenic derivatives [29].

At the Charité, where Robert Koch would later identify the tubercle bacillus (*Mycobacterium tuberculosis*) in 1882, the institution was already recognized for its rigorous treatment protocols and its pioneering role in the field of infectious disease medicine. Unfortunately, tuberculin therapy was only introduced nearly two decades after Eminovici’s death, in 1890. This treatment involved administering tuberculin (Koch’s fluid), an extract from cultures of *Mycobacterium tuberculosis*, injected into patients to stimulate an immune response, which was believed to aid in combating the disease [30]. The therapy consisted of subcutaneous injections of diluted Koch’s fluid, typically administered to areas such as between the shoulder blades or the lumbar region. This method became notable for producing localized inflammatory reactions in affected regions, such as facial lupus or scrofulous glands of the neck [31,32]. However, the treatment was not universally successful, with efficacy depending on the patient’s condition and the precision of application. Significant side effects and complications further limited its adoption as a standard therapy [30].

**Figure 11:**
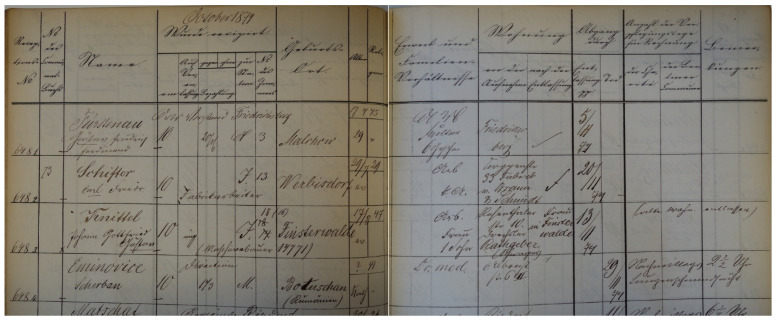
Official patient register of the Charité Hospital in Berlin from the period of Serban Eminovici’s admission *(images courtesy of Navena Widulin from the Berlin Museum of Medical History at the Charité)*.

Regarding Șerban Eminovici’s hospitalization at the Charité in October 1874, he was entered in the hospital’s official patient register, although no separate clinical records have survived. As shown in Figure 11, the register lists his identifying information on the last row: Patient number: 6484; Name: Eminovic Scherban; Admission date: 10 October 1874, by order of the director; Payment: 143, transferred to ward M (male); Place of birth: Botoșani (Romania); Age: ?–41; Religion: Catholic; Profession: Dr. Med. (doctor of medicine); Residence prior to admission: Albrechtstr. 6/II (second floor); Discharged by death: 29 November 1874; Notes: p.m. 2 ½ (2:30 p.m.), phthisis (pulmonary tuberculosis infection) (Figure 11).

By the time Dr. Șerban Eminovici was admitted to the renowned Charité Hospital in Berlin, his pulmonary disease had already reached a terminal stage. Regarding this, his brother, the poet Mihai Eminescu, wrote: “Șerban, as I had foreseen, is alienated and at the same time in a very advanced stage of lung disease. Even before entering the hospital, the secretary of the agency wrote to me that signs of mental alienation had been observed, which, increasing more and more, had reached a very high degree. His maintenance in the hospital costs eight napoleons per month, a sum I cannot afford” [9].

From this context, it can be inferred that tuberculosis had been complicated by a form of neurotuberculosis, as Șerban Eminovici showed signs of mental disturbance in the autumn of 1874 [6]. His condition deteriorated progressively, and the treatment available, although applied according to the most advanced protocols of the time, was insufficient to save his life, with death occurring on 29 November 1874 [4,10,33].

**Figure 12:**
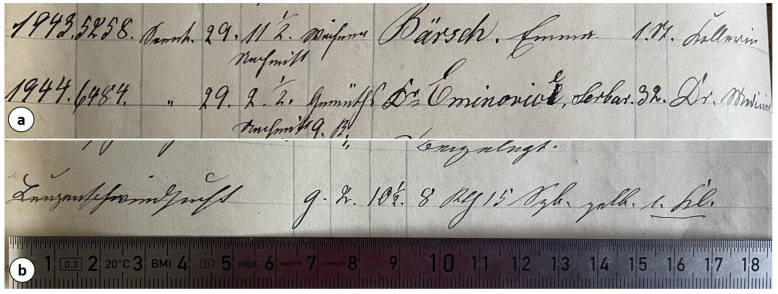
Detail from the death register (Leichenbuch) of the Charité Hospital, 1872–1874, showing the entry of “Dr. Eminovici Serban” (**a**) and the recorded cause of death, “Lungenschwindsucht” (tuberculosis), on 29 November at 2:30 p.m (**b**) *(images courtesy of Navena Widulin from the Berlin Museum of Medical History at the Charité)*.

In the death register (Leichenbuch) from the years 1872–1874 at the Charité Hospital in Berlin, the entry records that Șerban Eminovici died of “Lungenschwindsucht” (tuberculosis) on November 29 at 2:30 p.m. (Figure 12).

Subsequently, death certificate no. 458, dated 30 November 1874, recorded the date and time of Șerban Eminovici’s passing [10]. His death was officially announced by the Romanian Legation in Berlin at the end of November 1874. In a letter addressed by N. Crețulescu to the Romanian Ministry of Foreign Affairs, the demise of Dr. Șerban Eminovici, brother of Mihai Eminescu, was communicated along with the necessary measures regarding his personal effects, outstanding debts, hospital expenses, and burial arrangements [10].

Correspondence between the Romanian Legation in Berlin and the hospital administration reveals that Eminovici’s body was placed in the chapel of the Charité Hospital, located on Louisenstrasse no. 13. He was buried in the cemetery attached to the hospital on 20 November/2 December 1874 [10]. For the purpose of burial, Dr. Șerban Eminovici was declared Catholic; however, the funeral service was conducted according to the Orthodox rite by a priest summoned from the Russian chapel in Berlin, through the efforts of N. Crețulescu, the Romanian diplomatic envoy [10].

This loss had a profound impact on the Eminovici family, particularly on Mihai Eminescu, who at the time was in Germany and in close contact with his brother. Șerban Eminovici’s premature death represented a significant tragedy for the family, which had already been marked by numerous early deaths and mental health struggles among several members. His passing in Berlin occurred while he was successfully practicing medicine, being appreciated in contemporary scientific circles as a skilled physician and surgeon, and while he sought a cure for his progressive illness.

## 7. Comparative Context of Tuberculosis Treatment in 19th-Century Europe

In the second half of the nineteenth century, therapeutic approaches to pulmonary tuberculosis were largely uniform across Europe [17], relying on lifestyle-based interventions such as fresh air, rest, and high-calorie diets, in the absence of any curative pharmacological therapy. The first specialized sanatorium, established by Hermann Brehmer in Germany, became the prototype for similar institutions in Austria, Switzerland, and France, where patients were treated through aerotherapy and nutritional regimens [24].

Despite its reputation as one of Europe’s most advanced hospitals, the Charité Hospital in Berlin, where Dr. Șerban Eminovici was admitted in 1874, offered similar supportive care. At that time, the etiology of tuberculosis remained unknown, as Robert Koch would only identify the Mycobacterium tuberculosis eight years later, in 1882 [26]. Treatment at Charité reflected the best available practice (rest, cod liver oil, and hygienic regimen), but it was not curative.

Similar cases of intellectuals and physicians who succumbed to tuberculosis despite access to advanced medical care further illustrate the therapeutic limitations of the pre-antibiotic era. The writer Franz Kafka (1883–1924), who died of laryngeal tuberculosis, and the philosopher Eduard von Hartmann (1842–1906), who suffered from chronic pulmonary and skeletal tuberculosis, both experienced prolonged sanatorium treatment with no curative outcome. Like Șerban Eminovici, their trajectories highlight the pervasive reach of tuberculosis among Europe’s educated elites and the inability of even the most progressive medical institutions to prevent a fatal course.

## 8. Conclusions

In the 19th century, pulmonary tuberculosis was the leading cause of mortality in Europe, disproportionately affecting young people and socioeconomically disadvantaged groups in the absence of an effective etiological treatment. Dr. Șerban Eminovici (1841–1874), the elder brother of poet Mihai Eminescu, represents a significant case of the disease’s impact on the intellectual elites of the period. His medical education in Central Europe (Erlangen, Munich, Vienna, Berlin) and integration into prestigious scientific communities highlight the openness of Romanian elites to Western medical modernity. The progression of Eminovici’s illness from early balneary treatments to his terminal admission at the Charité Hospital in Berlin illustrates the limitations of pre-antibiotic therapeutic paradigms, focused on rest, nutrition, and aerotherapy, yet lacking curative options. The Eminovici case offers a dual historical perspective: on one hand, regarding the persistent vulnerability of young intellectuals to tuberculosis in the pre-Koch era; on the other, regarding the educational and professional mobility of Romanian elites within the European medical sphere.

## Figures and Tables

**Table 1:** Summary of the Eminovici Siblings: Year of Death, Age, Reported Illness, and Cause of Death.

No.	Name of Sibling	Year of Death	Age at Death (Years)	Reported Illness During Lifetime	Cause of Death
1	Ruxandra Eminovici	1845	<1 year	N/K	Unknown infant illness
2	Maria Eminovici	1848	8	N/K	Unknown infant illness
3	Vasile Eminovici	1857	4	N/K	Unknown infant illness
4	Ilie Eminovici	1867	21	—	Typhus
5	Iorgu Eminovici	1873	29	Mental disorder; Tuberculosis	Suicide (gunshot)
6	Șerban Eminovici	1874	33	Mental disorder; Tuberculosis	Pulmonary tuberculosis
7	Nicolae Eminovici	1884	41	Mental disorder	Suicide (gunshot)
8	Mihai Eminescu	1889	39	Mental disorder	Medical malpractice due to mercury poisoning (misdiagnosis of syphilis)
9	Harieta Eminovici	1889	35	Paraplegia (possible poliomyelitis); Tuberculosis	Cerebral stroke (apoplexy)
10	Aglaia Eminovici (Drogli)	1900	48	Tuberculosis	Pulmonary tuberculosis
11	Matei Eminovici	1929	73	Chronic pulmonary disease	Chronic pulmonary disease

N/K—not known.

## Data Availability

The original contributions presented in this study are included in the article. Further inquiries can be directed to the corresponding author.
